# Enhancing oocyte activation in women with ovarian failure: clinical outcomes of the Stem Cell Regenera study using G-CSF mobilization of peripheral blood stem cells and intraovarian injection of stem cell factor-enriched platelet rich plasma in real-world-practice

**DOI:** 10.18632/aging.206274

**Published:** 2025-06-27

**Authors:** Amparo Santamaria, Ana Ballester, Manuel Muñoz

**Affiliations:** 1 Reproductive Hematology Unit, IVIRMA Alicante Clinic, Ivi Clinics Alicante, Alicante 03003, Spain; 2 Department of Education and Research, European University Department, Ivi Clinics Alicante, Alicante 03003, Spain; 3 Gynecology Department, IVIRMA Alicante Clinics, Ivi Clinics Alicante, Alicante 03003, Spain

**Keywords:** Stem Cell Regenera, oocyte activation, ovarian regeneration, G-CSF, SCFE-PRP, ovarian failure

## Abstract

The study assesses the effectiveness and safety of the Stem Cell Regenera Treatment for oocyte activation in women with ovarian failure, including conditions such as Poor Ovarian Response (POR), Diminished Ovarian Reserve (DOR), and Premature Ovarian Insufficiency (POI). This retrospective observational study was conducted from January 2023 to December 2024 at the IVIRMA Alicante Clinics in Spain.

Women diagnosed with ovarian failure participated in the study, which involved mobilizing Hematopoietic Stem Cells from bone marrow into peripheral blood using granulocyte colony- stimulating factor (G-CSF). This was followed by an intraovarian injection of Stem Cell Factor- enriched Platelet Rich Plasma (SCFE-PRP).

The primary outcome measures were the rate of oocyte activation, leukocytes and stem cell count, and pregnancy rates. Oocyte activation was defined as an increase in total Antral Follicle Count of three or more follicles after treatment and/or at least a 20% rise in Anti-Müllerian Hormone levels. Safety was assessed based on adverse effects. Pregnancy rates were evaluated for both spontaneous gestation and following *in vitro* fertilization (IVF) treatment.

A total of 145 women participated: the overall activation rate was 68.28%, with 7.07% achieving spontaneous gestation and 14.14% achieving pregnancy following IVF. Mobilization of CD34+ cells was successful in all participants, with an average collection of 32.96 CD34+ cells/μl. No severe adverse effects were observed. The study concluded that the Stem Cell Regenera Treatment is effective and safe for oocyte activation in women with ovarian failure in real-world practice.

## INTRODUCTION

Recent advancements in regenerative medicine have provided new insights into the treatment of ovarian aging and ovarian failure. Women with conditions such as Poor Ovarian Response (POR) and Diminished Ovarian Reserve (DOR) face significant challenges in assisted reproduction [1–6]. These conditions result in a reduced natural conception rate, poor response to stimulation, and limited reproductive potential. Emerging evidence suggests that quiescent follicles in the ovaries can be activated through blood cell-based treatments, [5] including stem cell therapy and Platelet- Rich Plasma (PRP) administration [7–15].

In patients with cancer who undergo chemotherapy and bone marrow transplantation, there have been instances of regained ovarian function and spontaneous pregnancies. This occurrence has been associated with the mobilization of stem cells that reactivate ovarian function [16–19].

Research has indicated that various types of stem cells, including mesenchymal, endometrial, and hematopoietic stem cells, have the potential to restore ovarian function [20–22]. While most of these studies have been conducted on animal models, some have yielded encouraging results in human subjects. For instance, the ASCOT (Autologous Stem Cell Ovarian Transplantation) study reported notable improvements in ovarian reserve parameters and pregnancy rates following the direct injection of bone marrow stem cells into the ovary [23].

Also, Buigues et al. [24, 25] evaluated the noncellular components in different plasma sources enriched with stem cell-secreted factors. They found that plasma from women with POR treated with granulocyte colony-stimulating factor (G-CSF) improved pregnancy rates in mouse models. The regenerative properties of stem cell-enriched plasma were proved in human ovarian tissue, suggesting a paracrine signaling-based alternative for aging ovaries.

The experimental pilot study [26], known as the 4-step-ASCOT technique, evaluated ovarian reserve and reproductive outcomes in POI patients. This study involved mobilizing stem cells from bone marrow into peripheral blood, enriching plasma with stem cell factors, activating platelets, and injecting this enriched plasma directly into the ovaries. The results showed changes in the plasma proteome of POI women, with values like normal responders for a few months.

Based on these findings, the Stem Cell Regenera project was conducted. This treatment combines personalized G-CSF mobilization of Peripheral Blood hematopoietic Stem Cells with intraovarian injection of Stem Cell Factor-enriched (SCFE) PRP. The study aimed to assess the safety and effectiveness of the Stem Cell Regenera treatment for oocyte activation and ovarian regeneration in routine clinical practice.

## RESULTS

**Figure 1:**
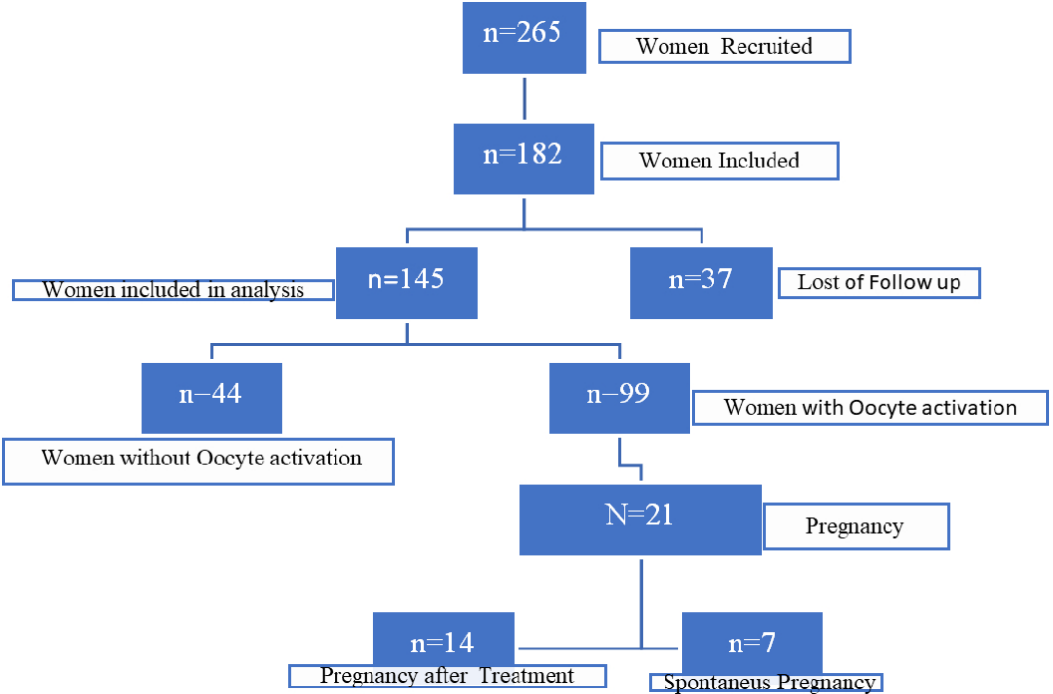
Flow chart depicting the participant selection, inclusion criteria, and outcomes in the Stem Cell Regenera study.

**Figure 2:**
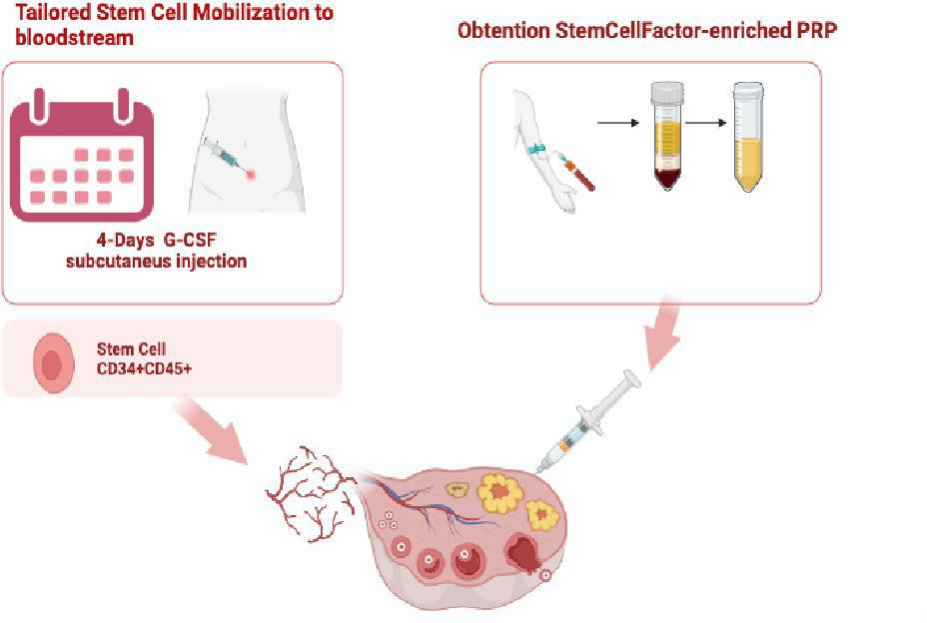
Schedule for the SC Regenera treatment.

We recruited 265 women for the study, and 182 met the inclusion criteria (see Figure 1). However, follow-up data was lost for 37 women See Figure 2. Therefore, a total of 145 women were included in the final analysis, with an average age of 39 years (ranging from 26 to 44 years).

The mean age was 39,1 years old. and ranged from 26 to 44 years, most participants identified as Caucasian (77.2%), followed by Arab (15.2%), Hispanic (4.8%), and other ethnicities.

The initial platelet count was 247,910 x 10 ^12^ /mm3 (range:107,000 - 455,000.) The mean white blood cell count was 6,025 x 10^9^/mm3 (range of 3,010 -10,220. x10^9^/mm3.and the mean hemoglobin was 13 g/dl (range 7.5-18.2). Demographic and characteristics of women are presented in Table 1.

**Table 1:** Characteristic and demographic data depending on oocyte activation.

	**Oocyte activation**		**P-value**
	**No**	**Yes**	
Age (Years-old, Range)	40 (26-44)	40 (31-44)	0.9
Hemoglobin at baseline(gr/dL) Mean, Range	13.1 (7.6-15.8)	13.6 (10,4-18,2)	0.2
Leucocytes at baseline (mm3) Mean, Range	5752 (3290-9900)	6151 (3010-10220)	0.2
Mean Platelets at baseline (mm3), Mean, Range	252522 (107000-455000)	245768 (150000-403000)	0.5

### Effectiveness of the stem cell regenera

The Stem Cell Regenera study aimed to evaluate the effectiveness and safety of stem cell mobilization using granulocyte colony-stimulating factor (G-CSF) and intraovarian injection of stem cell factor-enriched platelet-rich plasma (SCFE-PRP) for oocyte activation in women with poor ovarian response (POR) and diminished ovarian reserve (DOR). The age distribution of the patients was as follows: the mean age was 39.1 years, ranging from 26 to 44 years. Most participants identified as Caucasian (77.2%), followed by Arab (15.2%), Hispanic (4.8%), and other ethnicities 1. When comparing the age distribution between activated and non-activated groups, there was no significant difference in age (p=0.9). The overall oocyte activation rate was 68.28% of patients achieved OA (p<0.001) and the gestation rates observed were as follows: 7.07% of activated patients achieved spontaneous gestation (n=7), with a statistical significance of p<0.001. The overall gestation rate was 14.14% following treatment IVF (n=14), also with a statistical significance of p<0.001. None of the women without activation achieved pregnancy, either spontaneously or through IVF. See Table 2.

**Table 2:** Outcomes of stem cell regenera in ovocyte an activation gestational rate and leucocytes and CD34+ on fifth day.

**Activation**	**N**	Percentage (%)*	**Spontaneous gestation (%)**	**Gestation rate after IVF (%)**	Leucocytes (mm3)**	CD34+ / μ ***
No	46	31.72	0	0	37666 (20660- 58250)	26,5 (9-73)
Yes	99	68.28	7 (7.07)	14 (14.14)	36359 (14470-59670)	33,1 (2-140)

*P<0.00 **P<0.5 ***P<0.05.

### G-CSF Mobilization effectiveness evaluation

### Stem cell mobilization:

Mobilization of CD34+ cells was successful in all participants, with an average collection of 3000 CD34+ cells/mm3. The median number of CD34+ cells / μ in women who achieved oocyte activation was slightly higher than in those who did not (33.1 vs. 26.5, p=0.05). The median leukocyte count in women with oocyte activation was 36,359 x mm^3^ (range: 14,470-59,670). During the four-day treatment, nearly 30% of women needed adjustments to their G-CSF dosage.

This depended on their white blood cell count or the presence of moderate symptoms to achieve sufficient stem cell collection with minimal discomfort. Therefore, strict and daily monitoring was conducted. The effectiveness was analyzed by age groups, older and younger than 40 years old, and different diagnosis, but no differences were seen between either groups. The Pearson correlation coefficient was 0.465, showing a moderate positive relationship between white blood cells and CD34+ cells/μl. This suggests that as the value of white blood cells increases, the value of CD34+ cells/μl also tends to increase, with a statistical significance (p < 0.001) suggesting that this correlation is highly unlikely to be due to chance. In a clinical context, this moderate positive correlation implies that there is a consistent and meaningful relationship between the two variables. This can be useful for understanding how these variables behave together and can inform monitoring or treatment decisions. The significant correlation shows that the relationship is reliable and not random, which adds confidence to its clinical relevance.

The study aimed to establish a cutoff for CD34+ cells and leukocytes corresponding to a high rate of oocyte activation by analyzing different age groups and cutoff values. Women under 38 years of age exhibited a higher rate of activation compared to those aged 38 years and older. The distribution of CD34+ values between these two age groups showed no significant difference, and the distribution of leukocyte values also did not present a significant difference between the groups. A leukocyte count of 33,340 / mm3 was identified as the optimal cutoff point to maximize the difference between the true positive rate (TPR) and the false positive rate (FPR). Additionally, a CD34+ value of 5248.8 / mm3 was determined to be the best cutoff point for maximizing the difference between TPR and FPR. However, the area under the curve value of 0.487 indicates that CD34+ counts have a low capacity to differentiate between activation states in this study, potentially due to the small number of patients or other associated variables, or it may suggest that there is no definitive cutoff predicting activation.

Regression analysis suggested that variables such as age, leukocytes, and CD34+ significantly influence activation. Nonetheless, the model’s performance, given the limited patient sample, implies that other unconsidered factors like the patient’s baseline health, ovarian reserve, treatment protocol, and dosage of granulocyte colony-stimulating factor (G-CSF) may also contribute to the outcomes and require further statistical validation.

### G-CSF mobilization safety evaluation

- **Adverse Effects**: Most participants experienced only mild side effects, with no serious adverse effects reported. Specifically, 53.1% of women showed no symptoms, 32.8% experienced mild effects, and 14.1% encountered moderate to severe effects. Following mobilization treatment, all participants showed normalization of their peripheral blood counts. See Table 3.
- **Symptom Observation**: Daily observation of symptoms showed that most women experienced either no symptoms or only mild symptoms. The proportion of women experiencing mild symptoms increased from 8.28% on Day +1 to 58.6% on Day +4, while moderate symptoms were less common, and no symptoms decreased over time. See Table 3.

**Table 3:** Safety of the mobilization in terms of side effects associated with the treatment.

**Symptoms**	**Day +1**	**Day +2**	**Day +3**	**Day +4**
**Mild**	12 (8.28%)	59 (40.69%)	72 (49.66%)	85 (58.62%)
**Moderate**	0	12 (8.28%)	20 (13.79%)	17 (11.72%)
**None**	74 (51.03%)	74 (51.03%)	53 (36.55%)	43 (29.66%)

## DISCUSSION

### Main findings

This retrospective observational study evaluated the safety and effectiveness of the Stem Cell Regenera treatment for oocyte activation in 145 women diagnosed with ovarian failure, encompassing Poor Ovarian Response (POR), Diminished Ovarian Reserve (DOR), and Premature Ovarian Insufficiency (POI). The main finding was that 68.28% of patients achieved oocyte activation, defined as either an increase in total Antral Follicle Count (AFC) of three or more follicles after treatment or at least a 20% rise in Anti-Müllerian Hormone (AMH) levels. Furthermore, 7.07% of activated patients achieved spontaneous gestation, and 14.14% achieved pregnancy following IVF. Mobilization of CD34+ cells was successful in all participants, with an average collection of 3000 CD34+ cells/mm3, and no severe adverse effects were observed. A trend towards a higher median number of CD34+ cells in women who achieved oocyte activation compared to those who did not reach borderline statistical significance (p=0.05).

### Strengths and limitations

The strengths of this study include its evaluation of a relatively large cohort of women (n=145) undergoing Stem Cell Regenera treatment in a real-world clinical setting. The use of a standardized treatment protocol, involving G-CSF mobilization of peripheral blood hematopoietic stem cells and intraovarian injection of SCFE-PRP, allows for consistent assessment of outcomes. Furthermore, the clear definition of oocyte activation based on AFC and AMH levels provides objective measures of treatment effectiveness. It is noteworthy that our study primarily focused on oocyte activation as a direct biological endpoint, rather than solely on pregnancy outcomes. This choice of endpoint enabled us to reduce the required sample size while maintaining robust and clinically significant results. The inclusion of 145 women diagnosed with ovarian failure constitutes a substantial cohort for this specialized research area. Our sample size provided adequate statistical power to detect significant differences in key outcomes. These large effect sizes confirm the sufficiency of our cohort in identifying meaningful clinical differences. While the observation period may appear brief for comprehensively capturing all potential effects of the Stem Cell Regenera treatment on pregnancy rates, it is important to note that our study was designed specifically to evaluate both the immediate biological response (oocyte activation) and the short-term clinical outcomes (spontaneous and IVF-mediated pregnancies) following the intervention. Current evidence, coupled with our data [23], suggests that the mobilizing effect of G-CSF and intraovarian SCFE-PRP is most pronounced within the first 3–6 months post-treatment. This window is deemed optimal for assessing follicular activation and subsequent pregnancy attempts since most oocyte activation and pregnancies are anticipated to occur during this period. Our follow-up protocol ensured participants were closely monitored for these primary outcomes within the specified timeframe. Although a longer follow-up would be beneficial for evaluating cumulative pregnancy rates and long-term ovarian function, the chosen study duration was adequate for capturing the immediate and clinically pertinent effects of the treatment.

The mobilization of CD34+ cells was achieved in all participants without severe adverse effects, indicating the safety of the treatment protocol. G-CSF mobilization, which is commonly used in peripheral blood stem cell transplants, is a well-established procedure performed by hematologists globally. In this study, a hemotherapy specialist managed all aspects of G-CSF administration and patient monitoring, ensuring accurate dosing and prompt management of any adverse effects to maintain patient safety. Common side effects of G-CSF, such as filgrastim, are typically mild and transient, including bone pain, fatigue, mild headaches, or low-grade fever. Severe complications like allergic reactions are rare and other secondary effects are very rare in healthy donors. Extensive follow-up studies confirm these effects are mild and self-limiting with no long-term adverse outcomes. In our cohort, adverse events were mild and resolved spontaneously or with minimal intervention, aligning with broader literature. This supports the conclusion that G-CSF mobilization, when managed by experienced hematologists, is safe and well-tolerated. The risks associated with G-CSF mobilization are well understood, generally mild, and manageable through specialist-led care so that our findings reinforce its established safety.

The primary limitation of this study is its retrospective observational design, and the heterogenicity of the diagnosis. As a retrospective observational study, our design does not allow for definitive causal inference. However, our findings demonstrate a strong association between the intervention and significant improvements in both oocyte activation and pregnancy rates, with activation and pregnancy observed exclusively in the treated group.

Also, this study reflects real-world clinical practice. All participants had exhausted conventional fertility treatments and chose the Stem Cell Regenera protocol instead of oocyte donation. So that, randomizing patients to a non-treatment control group was not part of the study design.

Also, this study does not aim to establish causality but reports clinical outcomes and safety data observed in routine practice. It builds on our previous randomized study [23, 26], which established the approach’s rationale. We provide an observational account of the effectiveness and safety of this procedure rather than drawing definitive causal conclusions.

To address the lack of a control group, we applied stringent activation criteria and compared outcomes within the cohort, allowing an internal assessment of treatment response. Future studies with proper control arms are necessary to strengthen the evidence base, and this is a key priority for ongoing research.

Regarding the heterogeneity of our patient population and its potential impact on therapeutic response, we recognize that including women with Poor Ovarian Response (POR), Diminished Ovarian Reserve (DOR), and Premature Ovarian Insufficiency (POI) may introduce variability in clinical characteristics and potentially in treatment outcomes. Nonetheless, this heterogeneity accurately mirrors the real-world clinical setting in which the Stem Cell Regenera protocol is implemented. Our inclusion criteria were based on established ESHRE and POSEIDON definitions to ensure diagnostic clarity and minimize ambiguity.

Also to address the variability, we performed subgroup analyses according to diagnostic categories and found that, while the magnitude of response varied, the overall trend toward oocyte activation and pregnancy was consistent across groups. We also adjusted for relevant confounders such as age and baseline ovarian reserve in our statistical analyses to further account for differences within the population.

So that future studies focusing on more homogeneous patient groups could provide additional insights into which subpopulations benefit most from this treatment. Nonetheless, our findings offer valuable information on the effectiveness and safety of the Stem Cell Regenera protocol in a diverse, real-world population, which is representative of the patients encountered in daily clinical practice.

### Interpretation (in light of other evidence)

The findings of this study are consistent with previous research that suggests stem cell-based therapies may enhance ovarian function in women with ovarian failure. For instance, the ASCOT study reported improved ovarian reserve parameters and pregnancy rates following the direct injection of bone marrow stem cells into the ovary [23]. The Stem Cell Regenera treatment incorporates aspects of the 4-step-ASCOT technique [26], which includes mobilizing stem cells, enriching plasma with stem cell factors, activating platelets, and injecting the enriched plasma directly into the ovaries [14, 20–22]. While the Stem Cell Regenera treatment utilizes a tailored G- CSF mobilization protocol to mobilize peripheral blood stem cells, the fundamental principle of promoting ovarian regeneration through stem cell-mediated mechanisms remains the same. Additionally, there is no available information about the necessity of specific Stem Cell Peripheral bone marrow cells for better outcomes in ovarian failure 4-step-ASCOT technique or the data of efficacy and safety.

The results of this study should be considered in the context of existing literature, which contains conflicting findings regarding the efficacy of stem cell-based therapies for ovarian failure in clinical trials. It is noteworthy that none of these studies have published their results in real-world practice. While some studies have reported promising results, others have found no significant benefit or even adverse outcomes [1–15, 23–24). The heterogeneity of patient populations, treatment protocols, outcome measures, and study designs may account for these discrepancies.

The finding that a higher median number of CD34+ cells was seen in women with oocyte activation, while reaching borderline statistical significance, it suggests that the mobilization of stem cells may play a role in the treatment’s effectiveness. This is consistent with earlier research showing that CD34+ cells, a subset of hematopoietic stem cells, can promote angiogenesis and tissue regeneration [14]. However, the lack of statistical significance underscores the need for further investigation to elucidate the specific mechanisms through which stem cells may improve ovarian function. In comparison to these studies, Buigues et al. [26] evaluated the noncellular components in different plasma sources enriched with stem cell-secreted factors. They found that plasma from women with POR treated with granulocyte colony-stimulating factor (G-CSF) improved pregnancy rates in mouse models. The regenerative properties of stem cell-enriched plasma were proved in human ovarian tissue, suggesting a paracrine signaling-based alternative for aging ovaries.

Unlike oocyte donation, which serves as a therapeutic option for many women experiencing ovarian failure, the Stem Cell Regenera treatment potentially allows the use of the patient’s own genetic material.

## Conclusion

The Stem Cell Regenera treatment, which combines G-CSF mobilization of peripheral blood stem cells with intraovarian SCFE-PRP injections, shows promise for oocyte activation in about 70% of women with ovarian failure. It has also led to spontaneous gestations and pregnancies following IVF. This protocol successfully mobilized CD34+ cells in all participants without severe adverse effects. However, further research is needed to confirm these results.

## MATERIALS AND METHODS

From January 2023 to December 2024, we conducted a retrospective observational study of women diagnosed with Poor ovarian response (POR) and Diminished Ovarian Reserve (DOR), POI (premature ovarian insufficiency).

As inclusion criteria, we included women with Diminished Ovarian Reserve (DOR), Poor Ovarian Response (POR), and Premature Ovarian Insufficiency (POI). DOR refers to the decrease in ovarian reserve, with diagnostic criteria defined by Poseidon Groups 3 and 4. Group Poseidon 3 includes women under 35 years old with low ovarian reserve, characterized by Anti-Müllerian Hormone (AMH) levels below 1.2 ng/mL and an Antral Follicular Count (AFC) of fewer than 5 follicles, regardless of previous ovarian stimulation response. Group Poseidon 4 includes women aged 35 years or older with low ovarian reserve, also defined by AMH levels below 1.2 ng/mL and AFC of fewer than 5 follicles, regardless of previous ovarian stimulation response. POR is diagnosed according to the Bologna classification by ESHRE, which includes women aged 40 years or older or those with any indication of reduced ovarian reserve (low AMH, high FSH, low AFC) and a poor response in a previous IVF cycle (≤3 oocytes retrieved with standard stimulation). POI includes women who meet the ESHRE criteria for POI, characterized by the presence of menstrual disturbance defined as oligo/amenorrhea for at least 4 months, biochemical confirmation as evidenced by an elevated FSH level >25 IU/L on two occasions more than 4 weeks apart, or fluctuating POI when one of the above criteria is missing.

The exclusion criteria were age under 18, genetic or autoimmune ovarian insufficiency, clinical endometriosis, previous abdominal surgeries causing pelvic adhesions, non-visible ovaries inaccessible vaginally, positive serologies for HBsAg, HBcAc, HCV, HIV, syphilis, significant abnormalities in analytical results, and known intolerance or allergies to study product components.

### Methods

This study was conducted from January 2023 to December 2024 and involved women diagnosed with Poor Ovarian Response (POR), Diminished Ovarian Reserve (DOR), and Premature Ovarian Insufficiency (POI). The primary objective was to gather leukocytes and CD34+ cells for oocyte activation and examine their correlation with activation criteria.

The primary objective was to gather leukocytes and CD34+ cells for oocyte activation and examine their correlation with activation criteria. Effectiveness was measured by linking leukocytes and CD34+ cells on day five to the activation criteria, defined as a positive response when women showed an increase in Antral Follicle Count (AFC) by ultrasound of three or more follicles after treatment or at least a 20% rise in Anti-Müllerian Hormone (AMH) levels. Safety was determined based on adverse effects from G-CSF treatment, categorized as follows: none, mild (mild headache or bone pain without the need for pain medication), moderate (bone pain, headache, or other symptoms related to G-CSF requiring pain treatment or cessation of medication for 24 hours), and severe (bone pain or other related symptoms requiring intravenous pain treatment that led to discontinuation of G-CSF treatment).

The secondary objectives included evaluating the analytical parameters of each patient before and after G-CSF treatment. This involved quantifying red and white blood cells and platelets before and after treatment, measuring CD34+ prior to the PRP intraovarian injection, assessing follicular development through antral follicle count, determining the number of activated oocytes, and calculating the pregnancy rate.

These women chose the Stem Cell Regenera treatment instead of oocyte donation at IVIRMA Alicante Clinics, Spain. All study procedures were approved and conducted according to the Institutional Review Board of Hospital Universitario y Politécnico La Fe, Valencia, Spain and the Institutional Review Board and the Ethics Committee from University of Valencia, Valencia, Spain (2403-ALC-039-AS). Written Informed consent was obtained.

The Stem Cell Regenera treatment comprises a tailored mobilization of stem cells into the bloodstream using granulocyte colony-stimulating factor (G-CSF), followed by the intraovarian injection of SCFE PRP. The tailored mobilization protocol involves adjusting the G-CSF dosage daily based on leukocyte count and clinical symptoms reported by the patient. This customization ensures optimal mobilization of hematopoietic stem cells while minimizing adverse effects and maximizing the therapeutic benefits.

Figure 2 illustrates the schedule for the SC Regenera treatment. The first stage of the treatment was four-day mobilization with G-CSF Injection, starting with an SC dose of 10 mg/kg/d, adjusting daily based on leukocyte control. On the fifth day, stem cell circulating count and white blood cells were performed. Samples were analyzed by flow cytometry to quantify the CD34+ CD45+ population in the flow cytometer (DxFLex ®).

The second stage of treatment involved injecting SCFE-PRP into the ovarian marrow. The treatment consisted of an intraovarian injection of SCFE-PRP; administration to both ovaries in an ultrasound-guided process like that of an oocyte puncture, on the fifth day. The procedure to obtain SCFE-PRP-Endoret was according to a medical device developed by BTI Biotechnology Institute. (BTI®), the PRP was prepared according to a standard protocol (Endoret kit; BTI®). The final sample obtained was quantified to verify the platelet concentration and was injected transvaginal distributed equally between both ovaries. The total amount of SCFE-PRP injected ranged between 3.6 and 8 ml1.) [9].

### Statistical analysis

Statistical analysis was conducted using Chi-square and T-tests were used for median and range comparisons, nonparametric ROC curves and tests for equality of ROC areas across different age groups. Logistic regression was performed as well as Pearson Correlation. A p-value greater than 0.05 was considered statistically significant.

## References

[1] Na J, Kim GJ. Recent trends in stem cell therapy for premature ovarian insufficiency and its therapeutic potential: a review. J Ovarian Res. 2020; 13:74. 10.1186/s13048-020-00671-2 32576209 PMC7313218

[2] Ciccocioppo R, Cantore A, Chaimov D, Orlando G. Regenerative medicine: the red planet for clinicians. Intern Emerg Med. 2019; 14:911–21. 10.1007/s11739-019-02126-z 31203564

[3] Ferraretti AP, La Marca A, Fauser BC, Tarlatzis B, Nargund G, Gianaroli L, and ESHRE working group on Poor Ovarian Response Definition. ESHRE consensus on the definition of ‘poor response’ to ovarian stimulation for *in vitro* fertilization: the Bologna criteria. Hum Reprod. 2011; 26:1616–24. 10.1093/humrep/der092 21505041

[4] Webber L, Davies M, Anderson R, Bartlett J, Braat D, Cartwright B, Cifkova R, de Muinck Keizer-Schrama S, Hogervorst E, Janse F, Liao L, Vlaisavljevic V, Zillikens C, Vermeulen N, and European Society for Human Reproduction and Embryology (ESHRE) Guideline Group on POI. ESHRE Guideline: management of women with premature ovarian insufficiency. Hum Reprod. 2016; 31:926–37. 10.1093/humrep/dew027 27008889

[5] van Kasteren YM, Schoemaker J. Premature ovarian failure: a systematic review on therapeutic interventions to restore ovarian function and achieve pregnancy. Hum Reprod Update. 1999; 5:483–92. 10.1093/humupd/5.5.483 10582785

[6] Hansen KR, Craig LB, Zavy MT, Klein NA, Soules MR. Ovarian primordial and nongrowing follicle counts according to the Stages of Reproductive Aging Workshop (STRAW) staging system. Menopause. 2012; 19:164–71. 10.1097/gme.0b013e31823b0b2e 22189385 PMC3267013

[7] Huang Q, Liu B, Jiang R, Liao S, Wei Z, Bi Y, Liu X, Deng R, Jin Y, Tan Y, Yang Y, Qin A. G-CSF-mobilized peripheral blood mononuclear cells combined with platelet-rich plasma accelerate restoration of ovarian function in cyclophosphamide-induced POI rats†. Biol Reprod. 2019; 101:91–101. 10.1093/biolre/ioz077 31034039

[8] Pantos K, Simopoulou M, Pantou A, Rapani A, Tsioulou P, Nitsos N, Syrkos S, Pappas A, Koutsilieris M, Sfakianoudis K. A Case Series on Natural Conceptions Resulting in Ongoing Pregnancies in Menopausal and Prematurely Menopausal Women Following Platelet-Rich Plasma Treatment. Cell Transplant. 2019; 28:1333–40. 10.1177/0963689719859539 31271054 PMC6767896

[9] Cantero MM. Not all platelet-rich plasma are created equal. Curr Opin Obstet Gynecol. 2024; 36:118–23. 10.1097/GCO.0000000000000944 38324593

[10] Díaz-García C, Herraiz S, Pamplona L, Subirá J, Soriano MJ, Simon C, Seli E, Pellicer A. Follicular activation in women previously diagnosed with poor ovarian response: a randomized, controlled trial. Fertil Steril. 2022; 117:747–55. 10.1016/j.fertnstert.2021.12.034 35367015

[11] Herraiz S, Pellicer N, Romeu M, Pellicer A. Treatment potential of bone marrow-derived stem cells in women with diminished ovarian reserves and premature ovarian failure. Curr Opin Obstet Gynecol. 2019; 31:156–62. 10.1097/GCO.0000000000000531 30855290

[12] Shareghi-Oskoue O, Aghebati-Maleki L, Yousefi M. Transplantation of human umbilical cord mesenchymal stem cells to treat premature ovarian failure. Stem Cell Res Ther. 2021; 12:454. 10.1186/s13287-021-02529-w 34380572 PMC8359553

[13] Cui X, Jing X. Stem cell-based therapeutic potential in female ovarian aging and infertility. J Ovarian Res. 2024; 17:171. 10.1186/s13048-024-01492-3 39182123 PMC11344413

[14] Park HS, Ashour D, Elsharoud A, Chugh RM, Ismail N, El Andaloussi A, Al-Hendy A. Towards Cell free Therapy of Premature Ovarian Insufficiency: Human Bone Marrow Mesenchymal Stem Cells Secretome Enhances Angiogenesis in Human Ovarian Microvascular Endothelial Cells. HSOA J Stem Cells Res Dev Ther. 2019; 5:019. 10.24966/srdt-2060/100019 32494757 PMC7269190

[15] Sheikhansari G, Aghebati-Maleki L, Nouri M, Jadidi-Niaragh F, Yousefi M. Current approaches for the treatment of premature ovarian failure with stem cell therapy. Biomed Pharmacother. 2018; 102:254–62. 10.1016/j.biopha.2018.03.056 29567538

[16] Sameni HR, Seiri M, Safari M, Tabrizi Amjad MH, Khanmohammadi N, Zarbakhsh S. Bone Marrow Stromal Cells with the Granulocyte Colony-Stimulating Factor in the Management of Chemotherapy-Induced Ovarian Failure in a Rat Model. Iran J Med Sci. 2019; 44:135–45. 30936600 PMC6423433

[17] Lee HJ, Selesniemi K, Niikura Y, Niikura T, Klein R, Dombkowski DM, Tilly JL. Bone marrow transplantation generates immature oocytes and rescues long-term fertility in a preclinical mouse model of chemotherapy-induced premature ovarian failure. J Clin Oncol. 2007; 25:3198–204. 10.1200/JCO.2006.10.3028 17664466

[18] Liu J, Zhang H, Zhang Y, Li N, Wen Y, Cao F, Ai H, Xue X. Homing and restorative effects of bone marrow-derived mesenchymal stem cells on cisplatin injured ovaries in rats. Mol Cells. 2014; 37:865–72. 10.14348/molcells.2014.0145 25410907 PMC4275703

[19] Bao R, Xu P, Wang Y, Wang J, Xiao L, Li G, Zhang C. Bone marrow derived mesenchymal stem cells transplantation rescues premature ovarian insufficiency induced by chemotherapy. Gynecol Endocrinol. 2018; 34:320–6. 10.1080/09513590.2017.1393661 29073798

[20] Liesveld JL, Sharma N, Aljitawi OS. Stem cell homing: From physiology to therapeutics. Stem Cells. 2020; 38:1241–53. 10.1002/stem.3242 32526037

[21] Perlin JR, Sporrij A, Zon LI. Blood on the tracks: hematopoietic stem cell-endothelial cell interactions in homing and engraftment. J Mol Med (Berl). 2017; 95:809–19. 10.1007/s00109-017-1559-8 28702683 PMC5558790

[22] Yuan L, Huang W, Bi Y, Chen S, Wang X, Li T, Wei P, Du J, Zhao L, Liu B, Yang Y. G-CSF-mobilized peripheral blood mononuclear cells combined with platelet-rich plasma restored the ovarian function of aged rats. J Reprod Immunol. 2023; 158:103953. 10.1016/j.jri.2023.103953 37209460

[23] Herraiz S, Romeu M, Buigues A, Martínez S, Díaz-García C, Gómez-Seguí I, Martínez J, Pellicer N, Pellicer A. Autologous stem cell ovarian transplantation to increase reproductive potential in patients who are poor responders. Fertil Steril. 2018; 110:496–505.e1. 10.1016/j.fertnstert.2018.04.025 29960701

[24] Buigues A, Marchante M, de Miguel-Gómez L, Martinez J, Cervelló I, Pellicer A, Herraiz S. Stem cell-secreted factor therapy regenerates the ovarian niche and rescues follicles. Am J Obstet Gynecol. 2021; 225:65.e1–14. 10.1016/j.ajog.2021.01.023 33539826

[25] Buigues A, Ramírez-Martin N, Martínez J, Pellicer N, Meseguer M, Pellicer A, Herraiz S. Systemic changes induced by autologous stem cell ovarian transplant in plasma proteome of women with impaired ovarian reserves. Aging (Albany NY). 2023; 15:14553–73. 10.18632/aging.205400 38149997 PMC10781467

[26] Buigues A, Pellicer N, Martínez J, Ramírez-Martín N, Rodríguez-Hernández C, Blázquez P, Pellicer A, Herraiz S. The 4-step ASCOT technique modifies the plasma proteome of patients with premature ovarian insufficiency to resemble that of normoresponder women. Human Reproduction. 2024; 39(Supplement 1):deae108.1103. 10.1093/humrep/deae108.1103

